# Investigation of gene-gene interactions of clock genes for chronotype in a healthy Korean population

**DOI:** 10.5808/GI.2020.18.4.e38

**Published:** 2020-12-09

**Authors:** Mira Park, Soon Ae Kim, Jieun Shin, Eun-Jeong Joo

**Affiliations:** 1 Department of Preventive Medicine, Eulji University School of Medicine, Daejeon 34824, Korea; 2 Department of Pharmacology, Eulji University School of Medicine, Daejeon 34824, Korea; 3 Department of Liberal Arts, Woosuk University, Wanju 55338, Korea; 4 Department of Neuropsychiatry, Eulji University School of Medicine, Daejeon 34824, Korea; 5 Department of Psychiatry, Nowon Eulji Medical Center, Eulji University, Seoul 01830, Korea

**Keywords:** chronotype, circadian rhythm, clock genes, gene-gene interaction, quantitative multifactor dimensionality reduction

## Abstract

Chronotype is an important moderator of psychiatric illnesses, which seems to be controlled in some part by genetic factors. Clock genes are the most relevant genes for chronotype. In addition to the roles of individual genes, gene-gene interactions of clock genes substantially contribute to chronotype. We investigated genetic associations and gene-gene interactions of the clock genes *BHLHB2, CLOCK, CSNK1E, NR1D1, PER1, PER2, PER3*, and *TIMELESS* for chronotype in 1,293 healthy Korean individuals. Regression analysis was conducted to find associations between single nucleotide polymorphism (SNP) and chronotype. For gene-gene interaction analyses, the quantitative multifactor dimensionality reduction (QMDR) method, a nonparametric model-free method for quantitative phenotypes, were performed. No individual SNP or haplotype showed a significant association with chronotype by both regression analysis and single-locus model of QMDR. QMDR analysis identified *NR1D1* rs2314339 and *TIMELESS* rs4630333 as the best SNP pairs among two-locus interaction models associated with chronotype (cross-validation consistency [CVC] = 8/10, p = 0.041). For the three-locus interaction model, the SNP combination of *NR1D1* rs2314339, *TIMELESS* rs4630333, and *PER3* rs228669 showed the best results (CVC = 4/10, p < 0.001). However, because the mean differences between genotype combinations were minor, the clinical roles of clock gene interactions are unlikely to be critical.

## Introduction

Many biological processes have circadian rhythms with a cycle of approximately 24 hours. Such circadian rhythms appear at the cellular, molecular, tissue, and organ levels in humans. Both the suprachiasmatic nucleus, which is the internal master clock, and various environmental stimuli (e.g., the light-dark cycle) may play roles together in regulation of circadian rhythm. The sleep-wake cycle is one of the most distinct circadian rhythms. Humans have a preferred timing of sleeping and waking, the so-called chronotype. The degree of morning preference shows a continuum from morning type (with an advanced sleep phase) to evening type (with a delayed sleep phase) [1]. The chronotype is known to be determined in a complex manner by age, sex, and various environmental factors, including level of light exposure [2-4]. Developmental changes in chronotype occur; these include earlier preference during childhood, later preference in adolescence and early adulthood, and gradually earlier preference with advancing age [2].

Chronotype has been studied extensively in a number of ways. For convenience in many studies, chronotype has been measured subjectively and arbitrarily classified as morning, intermediate, and evening types, according to the Composite Scale of Morningness (CS) [5,6]. Chronotype shows a normal or near-normal distribution in the population; the heritability of chronotype has been shown to range between 21% and 52% [7]. Therefore, chronotype is presumably a polygenic quantitative trait [8,9]. Circadian rhythms are regulated by a set of circadian genes in mammals [9,10]. These circadian genes are also known as clock genes; they include *ADCYAP1, ARNTL, BHLHB2, BHLHB3, CLOCK, PER1, PER2, PER3, NR1D1, NPAS2, NR1D1, THRA, CSNK1D, CSNK1E, CRY2, RORA, RORB, TIMELESS*, and *VIP*. Notably, the list continues to grow due to discoveries in this field. Recently, three genome-wide association studies (GWASs) have been performed in people of European ancestry [11-13]. The results indicated meaningful overlap of genes that exhibited significant associations with chronotype. All three GWASs supported the association of four genes—*PER2, RGS16, FBXL13*, and *AK5*—with chronotype [14]. A meta-analysis of these three GWASs identified 351 loci associated with chronotype, of which 327 loci were novel and 24 loci had been reported in other GWASs [15].

GWASs can identify only common genetic variants, with small or modest effects for complex traits. Chronotype is a complex trait, which may be influenced by polygenes. A recent GWAS found only common genetic variants with small individual genetic effects on chronotypes. Only a subset of the significant variants found in that GWAS were known clock genes or have been suggested to function as clock genes. There were many other genetic variants without known functions as clock genes. Genetic control of chronotype must occur in multiple ways, with individual genotypic associations of polygenes, haplotypic associations of more crucial genes, and gene-gene interactions of multiple biologically related functional genes. Here, we investigated possible roles for gene-gene interactions of clock genes in chronotype in a Korean population.

## Methods

### Participants

The study population consisted of 1,293 unrelated healthy Korean individuals. These were the same individuals included as controls in our previous study of bipolar disorder [16]. They consisted mostly of college students, nurses, and public officials, who were recruited after a brief psychiatric interview. Potential participants were excluded if they reported a history of a psychotic disorder, mood disorder, anxiety disorder, substance use disorder, brain trauma, or intellectual disability. All participants were informed of the purpose and methods of the study and provided informed consent before enrollment. The Ethics Committee of Eulji General Hospital approved the study protocol (IRB No. 2016-08-009).

### Measurement of chronotype

Chronotype was measured using a self-reported questionnaire. The CS is a 13-item questionnaire, which assesses individual differences in the time of day a person prefers to carry out various activities; it classifies people as morning, intermediate, or evening types [5]. Three items are scored on a five-point scale from 1 to 5; the other 10 items are scored on a four-point scale, from 1 to 4. Higher scores indicate morning preference. All participants completed the CS questionnaire.

### Genotyping

The clock genes investigated in this study were *BHLHB2, CLOCK, CSNK1E, NR1D1, PER1, PER2, PER3*, and *TIMELESS*. These eight genes were analyzed for 19 different tag single nucleotide polymorphisms (SNPs) with minor allele frequencies exceeding 5% in Asian populations. DNA was extracted from blood and SNPs were genotyped using the TaqMan method (Applied Biosystems, Foster City, CA, USA). Table 1 presents a summary of the minor allele frequencies and chromosomal locations of the SNPs.

### Statistical analysis

Individual SNPs were examined for Hardy-Weinberg equilibrium; two SNPs violating Hardy-Weinberg equilibrium were removed. Each SNP association with CS score was analyzed by simple regression analysis. Haplotype association with CS was also analyzed by PLINK if more than two SNPs for each gene were included [17].

Gene-gene interactions were analyzed using the quantitative multifactor-dimensionality reduction (QMDR) method, an extension of the multifactor-dimensionality reduction (MDR) algorithm to work with quantitative or continuous phenotypes [18]. The MDR method is one a commonly used method for detection and characterization of high-order gene-gene or gene-environment interactions in case-control studies; this comprises a nonparametric combinatorial approach that reduces the number of dimensions [19]. For each multi-locus genotype combination, QMDR calculates the mean value of phenotype and compares it to the overall mean to determine the genotype combination is high risk or low risk. By pooling all the genotypes into either high-risk or low-risk groups, a new binary attribute is created. The t-test is used to compare the means between high and low risk groups using a t-test and t-statistic is used as a training score to choose the best model. In QMDR, the training and testing score are defined by t-test statistic. The training score is used to determine the best K-order interaction model. QMDR use 10-fold cross-validation and cross-validation consistencies (CVCs) of each model chosen are recorded. The best overall QMDR model is selected as that with the maximum testing score and highest cross-validation consistency. To estimate the p-values of the chosen model, empirical null distribution is used [18].

In this study, interactions of up to three loci were tested using 10-fold cross-validation in a search considering all possible SNP combinations. SNP combination with maximum CVC was considered the best model. p-values were determined empirically by 1,000-fold permutations of case and control labels.

## Results

The study population consisted of 481 male participants (37.2%) and 812 female participants (62.8%). Mean ages were 27.5 ± 8.3 years for male participants and 23.7 ± 3.5 years for female participants. Mean CS scores were 32.3 ± 6.4 for male participants and 29.7 ± 5.7 for female participants. The classification of chronotype using total CS score revealed 305 evening type participants (23.6%), 911 intermediate type participants (70.5%), and 77 morning type participants (6.0%) in the study population. The distributions of total CS score according to age and chronotype are shown in Table 2.

Two SNPs of *PER1* were excluded from further analysis because they were found to deviate from Hardy-Weinberg equilibrium. Ultimately, 17 SNPs of seven genes were analyzed. In regression analyses, no individual SNP showed a significant association with CS score (Table 3). There were no significant haplotype associations with CS score for any of the genes with more than two SNPs in this study (Table 4). On QMDR analyses, no single locus was found to be associated with chronotype, similar to the results of regression analysis. *NR1D1* rs2314339 and *TIMELESS* rs4630333 were significantly associated with chronotype in a two-locus model (CVC = 8/10, p = 0.041). In the three-locus models, *NR1D1* rs2314339, *TIMELESS* rs4630333, and *PER3* rs228669 showed the strongest association with chronotype (CVC = 4/10, p < 0.001). A summary of QMDR results with CVC > 1/10 is presented in Table 5.

## Discussion

We hypothesized that circadian genes play an important role in chronotype regulation and that there are gene-gene interaction effects on chronotype. We identified the best interaction models for two and three loci, as well as statistical significances of the best interaction models for chronotype in a Korean population, using the QMDR method and corresponding permutation test. However, it was difficult to conclude that there were clinically significant gene-gene interaction effects on chronotype based on our results, since the mean differences in total CS score between genotype combinations were minor.

Human chronotype is a heritable polygenic trait, and many groups have searched for the genes involved in chronotype. Many clock genes have been considered as strong candidate genes for chronotype because of their biological functions in circadian networks. There have been many single-gene association studies to identify genetic factors for chronotype. The first report of an association between chronotype and clock genes was related to the 3′-untranslated region of the *CLOCK* gene (rs1801260) [20]. This finding was not replicated in other populations [21-23], but was replicated in a Japanese population [24]. Multiple other clock genes have also been studied. The *PER3* gene was shown to be associated with delayed sleep phase syndrome. The studies thus far have focused on a variable number of tandem repeats region located in exon 18 of *PER3*, but the results of association analysis differed among studies. Specifically, a shorter allele was associated with delayed sleep phase syndrome [25]; a longer allele was also reportedly associated with delayed sleep phase syndrome [26] and was reported to have predictive value in response to sleep loss [27-29]. *PER1* and *PER2* were reported to be associated with advanced sleep phase syndrome in a British population [27,28], but not in a Japanese family [30]. Overall, the results were inconsistent and sometimes contradictory. Table 6 presents a summary of genetic association findings between clock genes and chronotype.

There have been several GWASs regarding chronotype in European populations using data from 23andMe and the UK Biobank [11-13,15]. There was a great deal of consistency across these studies, with nine genes identified in two of the three GWASs. Among these nine genes, four showed significant hits and were found in all three GWASs. Thus far, GWASs have been conducted only in populations of European ancestry. Emmanuel and von Schantz reported that six of 41 alleles identified earlier by GWASs in European participants (in the genes *RGS16, PER2*, and *AK5*, as well as between the genes *APH1A* and *CA14*) were absent from some non-European populations. This study underscores the ancestral diversity of circadian genetics and suggests that future studies should be performed in populations of East Asian ancestry [31].

The proteins encoded by various clock genes cooperate physically with each other and act as transcription factors. Combinations of polymorphisms in these genes may affect phenotype. Therefore, combined analysis of the effects of different clock genes may be more accurate and more revealing, compared to analyses of single genes. A few previous studies examined the effects of gene-gene interactions on chronotype. One study in Korean college students reported a significant interaction for CS score between *CLOCK* gene 3111 C/T and *GNB3* 825 C/T, according to regression analysis [32]. Later, the same group reported a genetic interaction for eveningness among *ARNTL, PER2*, and *GNB3*, according to MDR analysis [33]. Another study by Pedrazzoli et al. reported that a specific combination of polymorphisms in four clock genes was associated with diurnal preferences in a Brazilian population [34]. They chose four polymorphisms in four clock genes: *PER2, PER3, CLOCK*, and *BMAL1*. To the best of our knowledge, other than these studies, there have been no further analyses of the epistatic effects among clock genes for chronotype. Therefore, we attempted to identify gene-gene interactions among clock genes for chronotype in this study. We used QMDR to investigate gene-gene interactions to improve statistical power. We avoided subgrouping based on total CS score because grouping into morning, intermediate, or evening types could be arbitrary; moreover, we could address the quantitative score directly in our analysis. Multifactor dimensionality reduction is a common approach for identification of gene-gene interactions in case-control studies [19]. QMDR is an extension of MDR to handle quantitative phenotypes. Instead of comparing the case-control ratio of each multi-locus genotype to a fixed threshold as in MDR, QMDR compares the mean value of each multi-locus genotype to the overall mean [18].

In particular, gene-gene interactions among *NR1D1* rs2314339, *TIMELESS* rs4630333, and *PER3* rs228669 were significantly associated with chronotype in QMDR analyses in the present study. These SNPs did not show any associations as individual SNPs or haplotypes. *NR1D1* (nuclear receptor subfamily 1, group D, member 1) is the gene encoding REV-ERBα, located on chromosome 17q21.3. REV-ERBα suppresses the transcription of *BMAL1* mRNA [35,36], while BMAL1 activates REV-ERBα; this comprises a feedback loop of the mammalian circadian oscillator. *NR1D1* has been reported to show an association with chronotype in healthy Korean young adults [37]. Kang et al. [37] reported a significant association with rs12941497 of *NR1D1*, but no association with rs2314339, which showed significant gene-gene interactions with other SNPs in the present study. The *TIMELESS* gene was reportedly associated with morningness-eveningness in healthy university students [38]. The *PER3* gene showed conflicting results. Several positive associations for chronotype in European populations have been reported. In addition, negative findings were also reported for European populations [39-41] and Asian populations [42,43]. The circadian clock system consists mainly of transcription-translation feedback loops in the internal timekeeping clock. Heterodimers of *BMAL* (brain and muscle Arnt-like protein-1) and *CLOCK* (circadian locomotor output cycles kaput) activate the transcription of *PER* (period) and *CRY* (cryptochrome) genes. *CRY* and *PER* suppress transcriptional activity of *BMAL1/CLOCK* [54,55]. Because *NR1D1* is related to BMAL1 and *PER3* was reported to show a strong genetic interaction with *BMAL1*, our main finding supports this hypothesis. Unfortunately, no biological mechanism has been reported for chronotype that includes positive gene-gene interactions of all three genes identified in this study. Further studies are needed to understand the complicated biological molecular network of circadian clocks in humans.

Consistent and clear phenotyping is critical when performing genetic studies. We used CS score as a proxy for actual human diurnal preference. However, it evaluates both diurnal preference and sleep homeostasis. There are many ways to determine the timing of the circadian system (e.g., core body temperature monitoring, dim light melatonin onset, and actigraphy- or diary-based midpoint of sleep); self-reported diurnal preference is only a proxy method. Most GWASs have assessed chronotype using only a single question, such as “Are you naturally a night person or a morning person?” Non-precise phenotyping can produce unreliable significant findings that are unlikely to be replicated in subsequent studies [56]. Therefore, future phenotyping should include standardized self-reporting, clinical interviews, or objective assessment of sleep-wake periodicity, such as actigraphy. It is important to validate commonly used self-reported items. Comparisons between self-reported items and biological markers of circadian rhythms are needed to determine which questions are most closely associated with endogenous processes [14].

This study had some limitations that must be considered when interpreting our results. First, this study included only ethnically Korean individuals. Therefore, caution is needed when generalizing our results to other populations. As suggested in previous studies, chronotype and genes for chronotype are likely to differ according to ethnicity and/or between populations. Therefore, this study in a Korean population was necessary. Second, our participants were relatively young, with a mean age of 25 years. Because chronotype is affected by age, our results cannot be applied to other age groups. Third, because of resource limitations, we could include only 17 SNPs of seven circadian genes. Therefore, our results represent only a subset of the real-world epistatic interactions among clock genes. There are likely to be more complicated gene-gene interactions among circadian genes, as well as epistatic interactions between circadian genes and genes of other biological systems, which are directly and indirectly related to the circadian system.

We could not conclude that clock genes play a critical role in determining chronotype, although QMDR suggested significant gene-gene interactions. Further studies are needed to investigate gene-gene interactions of additional clock genes, especially with respect to SNPs that repeatedly show significant associations in multiple GWASs and studies in populations other than those of European ancestry.

## Figures and Tables

**Table 1.** SNPs of clock genes and minor allele frequency

Gene	SNP^a^	Base	Chr	Position	Function	MAF
*BHLHB2*	rs6442925	CT	3	4972191	Intron	0.047
	rs2137947	CT	3	4989276	Noncoding transcript variant	0.323
*CLOCK*	rs1801260	CT	4	55435202	3'-UTR	0.099
	rs3805148	AC	4	55440643	Intron	0.349
	rs12504300	CG	4	55482360	Intron	0.379
	rs4864542	CG	4	55487920	Intron	0.351
	rs12649507	AG	4	55514317	Intron	0.352
*CSNK1E*	rs135745	CG	22	38287631	None	0.223
	rs1534891	CT	22	38299094	Intron	0.093
	rs2075984	AC	22	38294883	Intron	0.408
*NR1D1*	rs2314339	CT	17	40096959	Intron	0.459
	rs2269457	AG	17	40098436	Intron	0.505
*PER2*	rs2304672	CG	2	238277948	5'-UTR	0.063
**	rs2304669	AG	2	238257022	Synonymous	0.116
*PER3 *	rs228669	AG	1	7809988	Synonymous	0.257
*TIMELESS*	rs4630333	AG	12	56443632	Intron	0.452
**	rs1082214	AG	12	56452706	Intron	0.095

SNP, single nucleotide polymorphism; Chr, chromosome; MAF, minor allele frequency; UTR, untranslated region.

a Total of 17 SNPs are remained after Hardy-Weinberg equilibrium test.

**Table 2.** CS score by age and chronotype distribution

Variable	Male	Female	Total
Age (y)	481	812	1,293
0‒9	1 (0.2)	0	1 (0.1)
10‒19	9 (1.9)	35 (4.3)	44 (3.4)
20‒29	147 (30.6)	361 (44.5)	508 (39.3)
30‒39	261 (54.3)	377 (46.4)	638 (49.3)
40‒49	61 (12.7)	39 (4.8)	100 (7.7)
50‒59	2 (0.4)	0	2 (0.2)
CS score	32.3 ± 6.4	29.7 ± 5.7	30.7 ± 6.1
Chronotype			
Evening type (≤26)	86 (17.9)	219 (27.0)	305 (23.6)
Intermediate type (27‒40)	346 (71.9)	565 (69.6)	911 (70.5)
Morning type (≥41)	49 (10.2)	28 (3.4)	77 (6.0)

Values are presented as number (%) or mean±SD.

CS, Composite Scale of Morningness.

**Table 3.** Individual SNP association analysis using simple regression

	Model 1				Model 2			
	Coefficient	SE	t	p-value	Coefficient	SE	t	p-value
*BHLHB2 *rs6442925CT	0.332	0.618	0.538	0.590	0.598	0.588	1.016	0.310
*BHLHB2 *rs2137947CT	0.052	0.255	0.205	0.838	0.033	0.243	0.134	0.893
*CLOCK *rs1801260CT	0.096	0.401	0.24	0.811	0.220	0.382	0.576	0.565
*CLOCK *rs3805148AC	0.181	0.248	0.731	0.465	0.214	0.236	0.905	0.366
*CLOCK *rs12504300CG	0.160	0.247	0.646	0.518	0.192	0.235	0.816	0.414
*CLOCK *rs4864542CG	0.160	0.247	0.646	0.518	0.192	0.235	0.816	0.414
*CLOCK *rs12649507AG	0.121	0.247	0.491	0.623	0.159	0.235	0.679	0.497
*CSNK1E *rs135745CG	‒0.262	0.319	‒0.822	0.411	‒0.307	0.304	‒1.009	0.313
*CSNK1E *rs1534891CT	0.550	0.403	1.364	0.173	0.357	0.384	0.929	0.353
*CSNK1E *rs2075984AC	‒0.159	0.238	‒0.666	0.506	‒0.121	0.227	‒0.534	0.594
*NR1D1 *rs2314339CT	‒0.229	0.238	‒0.964	0.335	‒0.259	0.226	-1.146	0.252
*NR1D1 *rs2269457AG	0.025	0.236	0.105	0.916	0.017	0.224	0.076	0.940
*PER2 *rs2304672CG	‒0.158	0.512	‒0.308	0.758	0.000	0.488	0.000	1.000
*PER2 *rs2304669AG	‒0.311	0.371	‒0.837	0.403	‒0.172	0.353	‒0.485	0.627
*PER3 *rs228669AG	0.482	0.266	1.809	0.071	0.4	0.254	1.577	0.115
*TIMELESS* rs4630333AG	‒0.127	0.241	‒0.527	0.598	‒0.176	0.23	‒0.768	0.443
*TIMELESS* rs1082214AG	‒0.372	0.433	-0.859	0.390	‒0.266	0.413	‒0.645	0.519

SNP, single nucleotide polymorphism; SE, standard error; Model 1, model without adjustment for age and sex; Model 2, with adjustment for age and sex.

**Table 4.** Haplotype association analysis

Gene	No. of SNPs	No. of haplotypes	F(df)	p-value
*BHLHB2*	2	4	0.124(3)	0.946
*CLOCK*	5	3	0.196(2)	0.822
*CSNK1E*	3	5	2.080(4)	0.082
*NRID*	2	4	0.513(3)	0.674
*PER2*	2	3	0.423(2)	0.655
*TIMELESS*	2	3	0.378(2)	0.685

No. of haplotypes indicates the number of common haplotypes (minor haplotype frequency ≥ 0.01). SNP, single nucleotide polymorphism.

**Table 5.** Summary of QMDR results having more than 1/10 of CVC

	CVC	Score^a^	p-value^b^
*NR1D1 *rs2314339CT	3/10	0.753	0.503
*TIMELESS *rs4630333AG	3/10	0.874	0.274
*BHLHB2 *rs2137947CT	2/10	0.760	0.492
*PER3 *rs228669AG	2/10	0.920	0.222
*NR1D1 *rs2314339,* TIMELESS *rs4630333**	8/10	1.193	0.041
*BHLHB2 *rs2137947CT,* TIMELESS *rs4630333	1/10	0.919	0.272
*NR1D1 *rs2314339CT,* PER3 *rs228669AG	1/10	1.054	0.117
*NR1D1 *rs2314339,* TIMELESS *rs4630333,* PER3 *rs228669**	4/10	1.741	<0.001
*CLOCK *rs12504300CG,* NR1D1 *rs2314339CT,* TIMELESS *rs4630333AG	3/10	0.897	0.303
*CSNK1E *rs2075984AC,* NR1D1 *rs2314339CT,* TIMELESS *rs4630333AG	2/10	0.915	0.277
*CSNK1E *rs2075984AC*, NR1D1 *rs2314339CT, *PER3 *rs228669AG	1/10	0.792	0.495

QMDR, quantitative multifactor dimensionality reduction; CVC, cross-validation consistency.

a Average testing score.

b Empirical p-value.

**Table 6.** Summary of SNP association findings between clock genes and chronotype

Gene	Polymorphism	Genetic region	Results	Population	Reference
*CLOCK*	rs1801260	3'-UTR	Positive association	American	[20]
			No association	British	[21]
			Positive association	Japanese	[24]
			No association	Brazilian	[23]
			No association	Korean	[32]
			No association	British	[39]
			No association	American	[44]
			No association	Italian	[45]
			No association	Korean	[33]
*ARNTL *	rs2278749	Intron	No association^a^	Korean	[33]
	rs7107287	Intron	No association	Poland	[38]
*ARNTL2*	rs922270	Intron	Positive association	British	[46]
*PER1*	rs2735611	Coding, synonymous	Positive association	British	[28]
*PER2*	rs2304672	5'-UTR	Positive association	Japanese	[30]
			No association	Italian	[48]
			No association	Korean	[47]
	rs934945	Missense	Positive association	Korean	[47]
			Positive association	Korean	[33]
	rs2304671	Coding, synonymous	Positive association	Japanese	[48]
	rs35333999	Missense	Positive association	British & American	[49]
*PER3*	rs57875989	Deletion/insertion	Positive association	British	[25]
	(VNTR)		Positive association	European^b^	[50]
			Positive association	British	[51]
			No association	British	[39]
			No association	Colombian	[41]
			No association	Norwegian	[40]
			No association	Han Chinese	[42]
			No association	Japanese	[43]
*TIMELESS*	rs2291738	Intron	Positive association	Poland	[38]
*NR1D1*	rs12941497	Intron	Positive association	Korean	[37]
*MTNR1B *	rs4753426	Promoter	Positive association	Brazilian	[52]
*GNB3*	rs5443	Coding, synonymous	Positive association	European^c^	[53]
			No association	Korean	[32]
			No association	Korean	[33]

SNP, single nucleotide polymorphism; UTR, untranslated region.

a Positive interaction with rs5443 of GNB3.

b European population in South Africa.

c European population in Sweden.

## References

[1] Roenneberg T, Merrow M (2007). Entrainment of the human circadian clock. Cold Spring Harb Symp Quant Biol.

[2] Duffy JF, Czeisler CA (2002). Age-related change in the relationship between circadian period, circadian phase, and diurnal preference in humans. Neurosci Lett.

[3] Allebrandt KV, Teder-Laving M, Kantermann T, Peters A, Campbell H, Rudan I (2014). Chronotype and sleep duration: the influence of season of assessment. Chronobiol Int.

[4] Fischer D, Lombardi DA, Marucci-Wellman H, Roenneberg T (2017). Chronotypes in the US: influence of age and sex. PLoS One.

[5] Horne JA, Ostberg O (1976). A self-assessment questionnaire to determine morningness-eveningness in human circadian rhythms. Int J Chronobiol.

[6] Smith CS, Reilly C, Midkiff K (1989). Evaluation of three circadian rhythm questionnaires with suggestions for an improved measure of morningness. J Appl Psychol.

[7] Barclay NL, Eley TC, Buysse DJ, Archer SN, Gregory AM (2010). Diurnal preference and sleep quality: same genes? A study of young adult twins. Chronobiol Int.

[8] McClung CA (2007). Circadian genes, rhythms and the biology of mood disorders. Pharmacol Ther.

[9] Lowrey PL, Takahashi JS (2011). Genetics of circadian rhythms in mammalian model organisms. Adv Genet.

[10] Dunlap JC (1999). Molecular bases for circadian clocks. Cell.

[11] Hu Y, Shmygelska A, Tran D, Eriksson N, Tung JY, Hinds DA (2016). GWAS of 89,283 individuals identifies genetic variants associated with self-reporting of being a morning person. Nat Commun.

[12] Jones SE, Tyrrell J, Wood AR, Beaumont RN, Ruth KS, Tuke MA (2016). Genome-wide association analyses in 128,266 individuals identifies new morningness and sleep duration loci. PLoS Genet.

[13] Lane JM, Vlasac I, Anderson SG, Kyle SD, Dixon WG, Bechtold DA (2016). Genome-wide association analysis identifies novel loci for chronotype in 100,420 individuals from the UK Biobank. Nat Commun.

[14] Kalmbach DA, Schneider LD, Cheung J, Bertrand SJ, Kariharan T, Pack AI (2017). Genetic basis of chronotype in humans: insights from three landmark GWAS. Sleep.

[15] Jones SE, Lane JM, Wood AR, van Hees VT, Tyrrell J, Beaumont RN (2019). Genome-wide association analyses of chronotype in 697,828 individuals provides insights into circadian rhythms. Nat Commun.

[16] Park M, Kim SA, Yee J, Shin J, Lee KY, Joo EJ (2019). Significant role of gene-gene interactions of clock genes in mood disorder. J Affect Disord.

[17] Purcell S, Neale B, Todd-Brown K, Thomas L, Ferreira MA, Bender D (2007). PLINK: a tool set for whole-genome association and population-based linkage analyses. Am J Hum Genet.

[18] Gui J, Moore JH, Williams SM, Andrews P, Hillege HL, van der Harst P (2013). A simple and computationally efficient approach to multifactor dimensionality reduction analysis of gene-gene interactions for quantitative traits. PLoS One.

[19] Ritchie MD, Hahn LW, Roodi N, Bailey LR, Dupont WD, Parl FF (2001). Multifactor-dimensionality reduction reveals high-order interactions among estrogen-metabolism genes in sporadic breast cancer. Am J Hum Genet.

[20] Katzenberg D, Young T, Finn L, Lin L, King DP, Takahashi JS (1998). A CLOCK polymorphism associated with human diurnal preference. Sleep.

[21] Robilliard DL, Archer SN, Arendt J, Lockley SW, Hack LM, English J (2002). The 3111 Clock gene polymorphism is not associated with sleep and circadian rhythmicity in phenotypically characterized human subjects. J Sleep Res.

[22] Adams CD, Jordahl KM, Copeland W, Mirick DK, Song X, Sather CL (2017). Nightshift work, chronotype, and genome-wide DNA methylation in blood. Epigenetics.

[23] Pedrazzoli M, Louzada FM, Pereira DS, Benedito-Silva AA, Lopez AR, Martynhak BJ (2007). Clock polymorphisms and circadian rhythms phenotypes in a sample of the Brazilian population. Chronobiol Int.

[24] Mishima K, Tozawa T, Satoh K, Saitoh H, Mishima Y (2005). The 3111T/C polymorphism of hClock is associated with evening preference and delayed sleep timing in a Japanese population sample. Am J Med Genet B Neuropsychiatr Genet.

[25] Archer SN, Robilliard DL, Skene DJ, Smits M, Williams A, Arendt J (2003). A length polymorphism in the circadian clock gene *Per3* is linked to delayed sleep phase syndrome and extreme diurnal preference. Sleep.

[26] Pereira DS, Tufik S, Louzada FM, Benedito-Silva AA, Lopez AR, Lemos NA (2005). Association of the length polymorphism in the human *Per3* gene with the delayed sleep-phase syndrome: does latitude have an influence upon it?. Sleep.

[27] Carpen JD, Archer SN, Skene DJ, Smits M, von Schantz M (2005). A single-nucleotide polymorphism in the 5'-untranslated region of the *hPER2* gene is associated with diurnal preference. J Sleep Res.

[28] Carpen JD, von Schantz M, Smits M, Skene DJ, Archer SN (2006). A silent polymorphism in the *PER1* gene associates with extreme diurnal preference in humans. J Hum Genet.

[29] Viola AU, Archer SN, Groeger JA, Skene DJ, Von Schantz M, Dijk DJ (2006). Polymorphism in the clock gene *PER3* predicts sleep structure and EEG power spectra. J Sleep Res.

[30] Satoh K, Mishima K, Inoue Y, Ebisawa T, Shimizu T (2003). Two pedigrees of familial advanced sleep phase syndrome in Japan. Sleep.

[31] Emmanuel P, von Schantz M (2018). Absence of morningness alleles in non-European populations. Chronobiol Int.

[32] Lee HJ, Paik JW, Kang SG, Lim SW, Kim L (2007). Allelic variants interaction of *CLOCK* gene and G-protein beta3 subunit gene with diurnal preference. Chronobiol Int.

[33] Song HM, Cho CH, Lee HJ, Moon JH, Kang SG, Yoon HK (2016). Association of *CLOCK, ARNTL, PER2*, and *GNB3* polymorphisms with diurnal preference in a Korean population. Chronobiol Int.

[34] Pedrazzoli M, Secolin R, Esteves LO, Pereira DS, Koike Bdel V, Louzada FM (2010). Interactions of polymorphisms in different clock genes associated with circadian phenotypes in humans. Genet Mol Biol.

[35] Liu AC, Tran HG, Zhang EE, Priest AA, Welsh DK, Kay SA (2008). Redundant function of REV-ERBalpha and beta and non-essential role for Bmal1 cycling in transcriptional regulation of intracellular circadian rhythms. PLoS Genet.

[36] Cho H, Zhao X, Hatori M, Yu RT, Barish GD, Lam MT (2012). Regulation of circadian behaviour and metabolism by REV-ERB-alpha and REV-ERB-beta. Nature.

[37] Kang JI, Park CI, Namkoong K, Kim SJ (2015). Associations between polymorphisms in the *NR1D1* gene encoding for nuclear receptor REV-ERBalpha and circadian typologies. Chronobiol Int.

[38] Jankowski KS, Dmitrzak-Weglarz M (2017). *ARNTL, CLOCK* and *PER3* polymorphisms: links with chronotype and affective dimensions. Chronobiol Int.

[39] Barclay NL, Eley TC, Mill J, Wong CC, Zavos HM, Archer SN (2011). Sleep quality and diurnal preference in a sample of young adults: associations with *5HTTLPR, PER3*, and *CLOCK* 3111. Am J Med Genet B Neuropsychiatr Genet.

[40] Osland TM, Bjorvatn BR, Steen VM, Pallesen S (2011). Association study of a variable-number tandem repeat polymorphism in the clock gene *PERIOD3* and chronotype in Norwegian university students. Chronobiol Int.

[41] Perea CS, Nino CL, Lopez-Leon S, Gutierrez R, Ojeda D, Arboleda H (2014). Study of a functional polymorphism in the *PER3* gene and diurnal preference in a Colombian sample. Open Neurol J.

[42] An H, Zhu Z, Zhou C, Geng P, Xu H, Wang H (2014). Chronotype and a *PERIOD3* variable number tandem repeat polymorphism in Han Chinese pilots. Int J Clin Exp Med.

[43] Hida A, Kitamura S, Kadotani H, Uchiyama M, Ebisawa T, Inoue Y (2018). Lack of association between *PER3* variable number tandem repeat and circadian rhythm sleep-wake disorders. Hum Genome Var.

[44] Chang AM, Buch AM, Bradstreet DS, Klements DJ, Duffy JF (2011). Human diurnal preference and circadian rhythmicity are not associated with the *CLOCK* 3111C/T gene polymorphism. J Biol Rhythms.

[45] Choub A, Mancuso M, Coppede F, LoGerfo A, Orsucci D, Petrozzi L (2011). *Clock* T3111C and *Per2* C111G SNPs do not influence circadian rhythmicity in healthy Italian population. Neurol Sci.

[46] Parsons MJ, Lester KJ, Barclay NL, Archer SN, Nolan PM, Eley TC (2014). Polymorphisms in the circadian expressed genes *PER3* and *ARNTL2* are associated with diurnal preference and GNbeta3 with sleep measures. J Sleep Res.

[47] Lee HJ, Kim L, Kang SG, Yoon HK, Choi JE, Park YM (2011). *PER2* variation is associated with diurnal preference in a Korean young population. Behav Genet.

[48] Matsuo I, Iijima N, Takumi K, Higo S, Aikawa S, Anzai M (2016). Characterization of sevoflurane effects on Per2 expression using ex vivo bioluminescence imaging of the suprachiasmatic nucleus in transgenic rats. Neurosci Res.

[49] Chang AM, Duffy JF, Buxton OM, Lane JM, Aeschbach D, Anderson C (2019). Chronotype genetic variant in *PER2* is associated with intrinsic Circadian period in humans. Sci Rep.

[50] Kunorozva L, Stephenson KJ, Rae DE, Roden LC (2012). Chronotype and *PERIOD3* variable number tandem repeat polymorphism in individual sports athletes. Chronobiol Int.

[51] Lazar AS, Slak A, Lo JC, Santhi N, von Schantz M, Archer SN (2012). Sleep, diurnal preference, health, and psychological well-being: a prospective single-allelic-variation study. Chronobiol Int.

[52] Silva A, Santos MJ, Koike BD, Moreira MS, Gitai DL, de Miranda Coelho JA (2019). Melatonin receptor 1B -1193T>C polymorphism is associated with diurnal preference and sleep habits. Sleep Med.

[53] Johansson C, Willeit M, Aron L, Smedh C, Ekholm J, Paunio T (2004). Seasonal affective disorder and the G-protein beta-3-subunit C825T polymorphism. Biol Psychiatry.

[54] Bass J, Takahashi JS (2010). Circadian integration of metabolism and energetics. Science.

[55] Feng D, Lazar MA (2012). Clocks, metabolism, and the epigenome. Mol Cell.

[56] Ioannidis JP, Thomas G, Daly MJ (2009). Validating, augmenting and refining genome-wide association signals. Nat Rev Genet.

